# LAMB3 Promotes Myofibrogenesis and Cytoskeletal Reorganization in Endometrial Stromal Cells via the RhoA/ROCK1/MYL9 Pathway

**DOI:** 10.1007/s12013-023-01186-5

**Published:** 2023-10-06

**Authors:** Xiaomei Qin, Bin Zeng, Suren R. Sooranna, Mujun Li

**Affiliations:** 1 Gynecology Section, Department of Obstetrics and Gynecology, The First Affiliated Hospital, Guangxi Medical University, 530000 Nanning, China; 2 Reproductive Medical Center, The First Affiliated Hospital, Guangxi Medical University, 530000 Nanning, China; 3 Department of Metabolism, Digestion and Reproduction Faculty of Medicine Imperial College London Chelsea & Westminster Hospital, London, SW10 9NH UK; 4 Life Science and Clinical Research Center, Youjiang Medical University for Nationalities, Baise, China

**Keywords:** LAMB3, Myofibrogenesis, Asherman’s syndrome, RhoA/ROCK1/MYL9, Cytoskeletal Reorganization

## Abstract

LAMB3, a major extracellular matrix and basal membrane component, is involved in wound healing. We aimed to understand its role in Asherman’s syndrome (AS), which is associated with infertility, by using bioinformatics analysis and cultured endometrial stromal cells (ESCs). MRNAs extracted from tissues obtained from control subjects and patients with severe intrauterine adhesion were sequenced and subjected to bioinformatics analysis and the RhoA/ROCK1/MYL9 pathway was implicated and this subsequently studied using cultured primary ESCs. The effects of overexpression and knockdown and activation and inhibition of LAMB3 on the mesenchymal to myofibroblastic phenotypic transformation of ECCs were assessed using PCR and western blot analysis. Phalloidin was used to localize the actin cytoskeletal proteins. Silencing of LAMB3 reversed the TGF-β-induced ESC myofibroblast phenotype conversion, whereas overexpression of LAMB3 promoted this process. Activation and silencing of LAMB3 led to remodeling of the ESC cytoskeleton. Overexpression and silencing of LAMB3 caused activation and inhibition of ESCs, respectively. Y-27632 and LPA reversed the activation and inhibition of the RhoA/ROCK1/MYL9 pathway after overexpression and silencing, respectively. These results suggest that LAMB3 can regulate ESC fibrosis transformation and cytoskeleton remodeling via the RhoA/ROCK1/MYL9 pathway. This study provides a potential new target for gene therapy and drug intervention of AS.

## Introduction

Asherman’s syndrome (AS) is characterized by fibrosis of the endometrium, due to trauma and infection, resulting in a replacement of the vascularized stroma with an avascular fibroblastic as well as a spindle-shaped myofibroblastic cell population [1–3]. This condition may go unrecognized in women who are not trying to conceive, but it is found in 1.5% of women evaluated with a hysterosalpingogram for infertility and in 5–39% of those who have experienced recurrent miscarriages. AS occurs in 31% of women after the initial hysteroscopic resection of leiomyoma, and up to 46% after the second resection [4, 5]. The incidence of AS was 1.6% in 2546 patients who had surgical abortions (uterine evacuation and curettage) before the 20th week gestation [6]. The pathophysiological changes associated with AS include an inactive single-layered cuboidal epithelium that is unresponsive to hormonal stimulation and an inability to form cyclic alterations. In addition, there is excessive matrix accumulation and deposition in the stroma, as well as fibrous adhesions [7, 8]. Clinical manifestations include menstrual disorders, amenorrhea, reduced menstrual flow, severe cyclic abdominal pain, infertility, abortion, fetal death, previa placenta and placental implantation, which can seriously jeopardize the reproductive health of women worldwide [9].

Muscle-derived fibroblasts were first discovered to be involved in the wound healing process where they play a key role in the formation of fibrotic scarring [10]. They express alpha-smooth muscle actin (α-SMA) and their presence lead to an increase in cell contractility, collagen production as well as myofibroblastic differentiation [11, 12]. Muscle fibroblasts, through an inflammatory reaction in and around damaged tissues, produce a large amounts of extracellular matrix components, including collagen types I and III, glycoproteins and proteoglycans, which all play important roles in the pathogenesis of fibrosis [13–15]. Changes in the cellular ultrastructure also play an important role this process. Studies have revealed a close relationship between the cytoskeleton and fibrosis in the heart [16], liver [17] and vas deferens [18]. The integrity of the cytoskeleton is essential for maintaining their normal physiological functions, and only F-actin has a biologically active role. It is involved in regulating cell migration, adhesion, stress fiber formation, material transportation, intermembrane information transmission [19, 20] as well as the maintenance of cellular morphology [21, 22].

This study was undertaken to evaluate the molecular mechanisms involved in myometrial fibrosis with a view to helping patients with infertility. We used bioinformatic analysis and in vitro cultures of endometrial stromal cells (ESCs) to investigate the regulation of myofibroblastic transformation of ESCs through the RhoA/ROCK1/MYL9 pathway with a view to finding potential drug targets to treat patients with AS.

## Materials and Methods

### Human Endometrium

The Ethics Committee of the First Affiliated Hospital of Guangxi Medical University approved the protocol (H-36683) for collection of the endometrial samples from 22 patients undergoing hysteroscopy. Informed consent was obtained from all subjects involved in the study. The patients were divided into two groups: an IUA group, which consisted of patients with severe intrauterine adhesion (IUA, grade IV–V) according to the classification and diagnostic criteria developed by the European Society of Gynecological Endoscopy [23], and a Control group. Each group consisted of 11 patients. The control group was composed of patients undergoing hysteroscopy for other reasons such as infertility or uterine septum and they showed no signs of IUA. All patients who underwent hysteroscopy had an endometrial tissue sample taken at 3–7 days after menstrual cleanliness, and the specimens were placed in cryogenic tubes and rapidly frozen in liquid nitrogen.

### Transcriptome Sequencing

Transcriptome analysis was performed on 3 patients from the IUA group and 3 patients from the control group. 50–100 mg of tissues were rapidly cut into small pieces with micro-scissors and microRNAs were extracted using an RNAmisi microRNA Rapid Extraction Kit (RN0501, Aidlab Biotechnologies, Beijing, China) according to the manufacturer’s instructions. For the RNA sequencing protocol, transcriptome libraries were prepared from mRNA of 3 tissue samples which were enriched using magnetic beads containing Oligo (dT). Subsequently, a fragmentation buffer was added to split the mRNAs into short fragments. Using mRNA as a template, a single strand cDNA was synthesized using six random primers. Then, buffer, dNTPs, RNase H, and DNA polymerase I were added to synthesize a double stranded cDNAs which were purified using AMPure P beans or QiaQuick PCR kit. After purification, they were subjected to end repair, A-tailed addition and sequencing connections, followed by fragment size selection and PCR enrichment to obtain the cDNA library. This was quantified and the size was determined using an Agilent 2100 column. 3 and cBot was used to generate clusters. After this, a dual ended sequencing program (PE) was run on the HiSeq sequencing platform to obtain 150 bp of dual ended sequencing reads. The samples obtained from the tissues of the IUA and control groups were submitted to Annoroad Gene Technology Corporation (Beijing) for RNA quality examination, library preparation and RNA sequencing. After cluster generation, the libraries were sequenced on an Illumina platform and 150 bp paired-end reads were generated.

### Bioinformatic Analysis

We used Bowtie (v 0.12.7) [24] to map the RNA-seq reads to the human genome hg19 and splice junctions. The read counts were computed for each gene through HTseq [25]. Differential expression of genes was analyzed with the R software package (http://www.R-project.org) and then the Bioconductor R package edgeR [26]. A heatmap from the differential expression levels was generated, and the genes with an adjusted *p* value < 0.05 and greater than a log_2_ (2-fold change) expression were considered to be differentially expressed. The cluster profiler program was used for Gene Ontology (GO) term enrichment. KEGG enrichment analysis was performed on the DEGs by using the David online database (https://david.ncifcrf.gov/summary.jsp).

### Primary Culture of ESCs

The collection of endometrial tissues for primary ESC cell cultures was also conducted based on protocol H-36683 that was approved by the Ethics Review Committee of the First Affiliated Hospital of Guangxi Medical University. Informed consent was obtained from all subjects involved in the study. The endometrial tissue samples were washed multiple times with PBS solution containing 0.1% penicillin-streptomycin to remove excess blood. They were cut into pieces with approximate volumes of 1.0 mm^3^, and then transferred to centrifuge tubes. After addition of 0.01% IV type collagenase at a ratio of 3:1, the tissues were digested at 37 °C in a constant temperature chamber with slow oscillation for approximately 30 min. The cell pellet was collected after centrifugation and this was re-suspended in DMEM/F12 culture medium. The cell suspension was pass 100- and 400-mesh nylon sieves, respectively, and the filtrate containing the ESCs was collected by further centrifugation. The pellet was the re-suspended in complete medium and dispersed. The cells were incubated in 6-well plates (Sigma, Shanghai, China) at 37 °C in a 5% CO2 incubator. 2 h and 6 h later, the cell morphology and apposition were observed, and the culture medium was replaced after 24 h. Primary human endometrial stromal cells were cultured in a 10% fetal calf serum (A3161001C, Gibco) containing dual antibody DMEM/F12 (C11330500BT, Gibco) complete medium and 1% penicillin-streptomycin solution (15140148; Gibco) at 37 °C in a 5% CO2 incubator. Culture media were subsequently changed every 2–3 days.

The cells were identified using immunocytochemistry and the mesenchymal cells were vimentin-positive. Cells were also incubated with the inhibitor, Y-27632 (50 µM, MCE, USA) and the activator, lysophosphatidic acid (LPA, 10 µM, Sigma, USA), respectively for 48 h.

### Generation of Stable Knockdown and Overexpressed Cells

Stable knockdown and overexpression of LAMB3 was achieved by using lentiviruses. We inserted human LAMB3 (GenBank accession number NM_000228) into the GV492 lentiviral vectors (Jikai, Shanghai, China) in order to silence and overexpress the expression of GAL1. The sequence information with respect to the lentiviruses used in this study are listed in Supplementary Table 1. The products of three human-specific LAMB3 knockdown sequences (sh1-LAMB3, sh2-LAMB3 and sh3-LAMB3) and one overexpressed sequence (OE-LAMB3) were verified by qRT-PCR and western blotting analysis. The lentiviral vectors were transfected into the ESCs at a multiplicity of infection of 50 in the presence of 2 μg/mL puromycin (Sigma-Aldrich, St. Louis, MO, USA). The pre-and post-transfection protocols used for ESCs are detailed in Supplementary Figs. 1 and 2, respectively.

### Immunohistochemical (IHC) Analysis

IHC staining was used to detect the expression and tissue localization of LAMB3 in the endometrium from IUA patients and those in the control group. 3 mm thick sections were cut from paraffin-embedded endometrial tissues. After being dewaxed and hydrated, the sections were subjected to antigen retrieval with 10 mM citrate buffer (pH 6.0) in a 700 W microwave for two 8 min cycles. Inactivation of endogenous peroxidase was achieved by 10 min incubation at room temperature with 3% H_2_O_2_ and 1% goat serum and then blocking was carried out for 1 h. The sections were incubated with antibody specific to LAMB3 (Abmart, Shanghai, China) at 4 °C for 18 h. After washing with PBS, the sections were incubated with an HRP-conjugated secondary antibody (Zhongshan, Beijing, China) for 30 min. The cultured ESCs were also fixed with 4% paraformaldehyde and incubated with vimentin (Proteintech, Wuhan, China) and CK19 (Proteintech) primary antibody respectively. The protocol for identification of ESCs using IHC is detailed in Supplementary Fig. 3. After washing, the peroxidase label was developed with DAB and this was followed by staining with hematoxylin. The results obtained were analyzed by using Image J software.

### Real-time Quantitative PCR (RT-qPCR)

Total RNA was extracted using column purification by addition of a commercial reagent (Aidlab, Beijing, China). cDNA was synthesized using a PrimeScript RT reagent kit (TaKaRa, Tokyo, Japan) according to the manufacturer’s instructions. RT-qPCR determinations were performed with specific primers and a PCR Master Mix (TaKaRa, Tokyo, Japan). The primers used are listed in Supplementary Table 2. The qRT-PCR program was set with the following parameters: 95 °C for 30 s, 40 cycles of 95 °C for 5 s, 60 °C for 34 s, 95 °C for 15 s and 60 °C for 60 s. The primer standard curves were plotted, the amplification efficiency was calculated and the Ct values were obtained by using the internal software of the 7500 Fast Real-Time PCR System (ABI, USA). Each of the reactions was performed in triplicate. The primer efficiency was determined before initiation of measurements. The specificity of the RT-qPCR products was verified by melting curve analysis. The amplification products were analyzed by electrophoresis on agarose gel for band size consistency after RT-qPCR was terminated. The 2-ΔΔCt method was used to calculate the expression levels of the target genes.

### Western Blotting Analysis

Cells were lysed in RIPA buffer (150 μL/cm3) prior to SDS-PAGE electrophoresis. The proteins were transferred from the gel to methanol-activated PVDF membranes at 110 V for 2 h. The membranes were washed twice with 1X TBST and then incubated with 5% non-fat milk for 1 h at room temperature in order to block nonspecific binding. Next, they were incubated with primary antibodies overnight at 4 °C. The primary antibodies used were as follows: anti-collagen I (ab138492, Abcam), anti-GAPDH (ab181602, Abcam), anti-alpha smooth muscle actin (ab124964, Abcam), anti-LAMB3 (TD2381S, Abmart), anti-RhoA (ab187027, abcam), anti-MYL9 (ab191393, Abcam) and anti-ROCK1 (ab134181, Abcam). After washing, the blotted membranes were then incubated with the appropriate secondary antibodies conjugated to horseradish peroxidase. The protein bands were then visualized by using an enhanced chemiluminescence detection system (ECL Substrate Kit, Abcam).

### Phalloidin Labeling Assay

Using a cell density of 5 × 10^3^ cells/coverslip, uterine stromal cells were seeded into 6-well plates and grown for 24 h to reach 50% confluence. The cells were then fixed with 4% paraformaldehyde solution. They were then permeabilized with 0.5% Triton X-100 (T8200, Solarbio, Beijing) solution and labeled with rhodamine phalloidin (40734ES75, Yisheng, Shanghai), and incubated in the dark for 30 min. Anti-fluorescence quenching blockers were used to block the cell nuclei from being re-stained. Fluorescence was observed under a confocal microscope with three channels. These were an FITC excitation/emission filter (Ex/Em = 496/516 nm), a TRITC excitation/emission filter (Ex/Em = 545/570 nm) and a DAPI excitation/emission filter (Ex/Em = 364/454 nm).

### Statistical Analysis

All the experimental results are expressed as means ± SDs of triplicate determinations. Statistical analyses were performed with the student’s *t* test using GraphPad Prism 8.0 (GraphPad Software). The difference was considered statistically significant when the *P* value was <0.05.

## Results

### RNA Sequencing Gene Expression

**Fig. 1:**
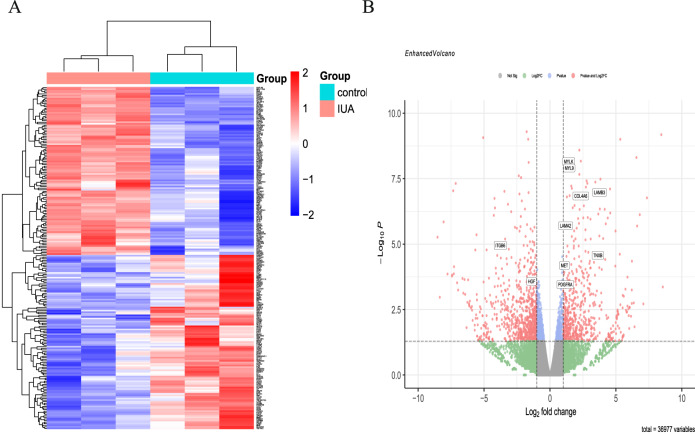
The expression profiles of the IUA and control groups. **A** A heatmap of the upregulated and downregulated genes with at least twofold difference between the IUA and control groups; **B** A volcano plot depicting the log_2_ FC plotted against log-normalized *p* values. The dotted horizontal line indicates the negative logarithmic adjusted *p* value (0.05) cut-off. The dotted vertical lines indicate the cut-off values of log_2_ FC. The red dots illustrate the differentially expressed genes and the ones named represent those associated with the RhoA/ROCK1/MYL9 pathway

By satisfying both the conditions of *P* ≤ 0.05 and |Fold change | ≥ 2, the genes with more than twofold difference were selected for further analysis. A comparison between the IUA and control groups revealed 399 DEGs, including 178 and 221 that were downregulated and upregulated, respectively. The upregulated and downregulated genes are depicted in a heatmap and volcano plot in Fig. 1A, B, respectively.

### KEGG Signaling Pathway Analysis

**Fig. 2:**
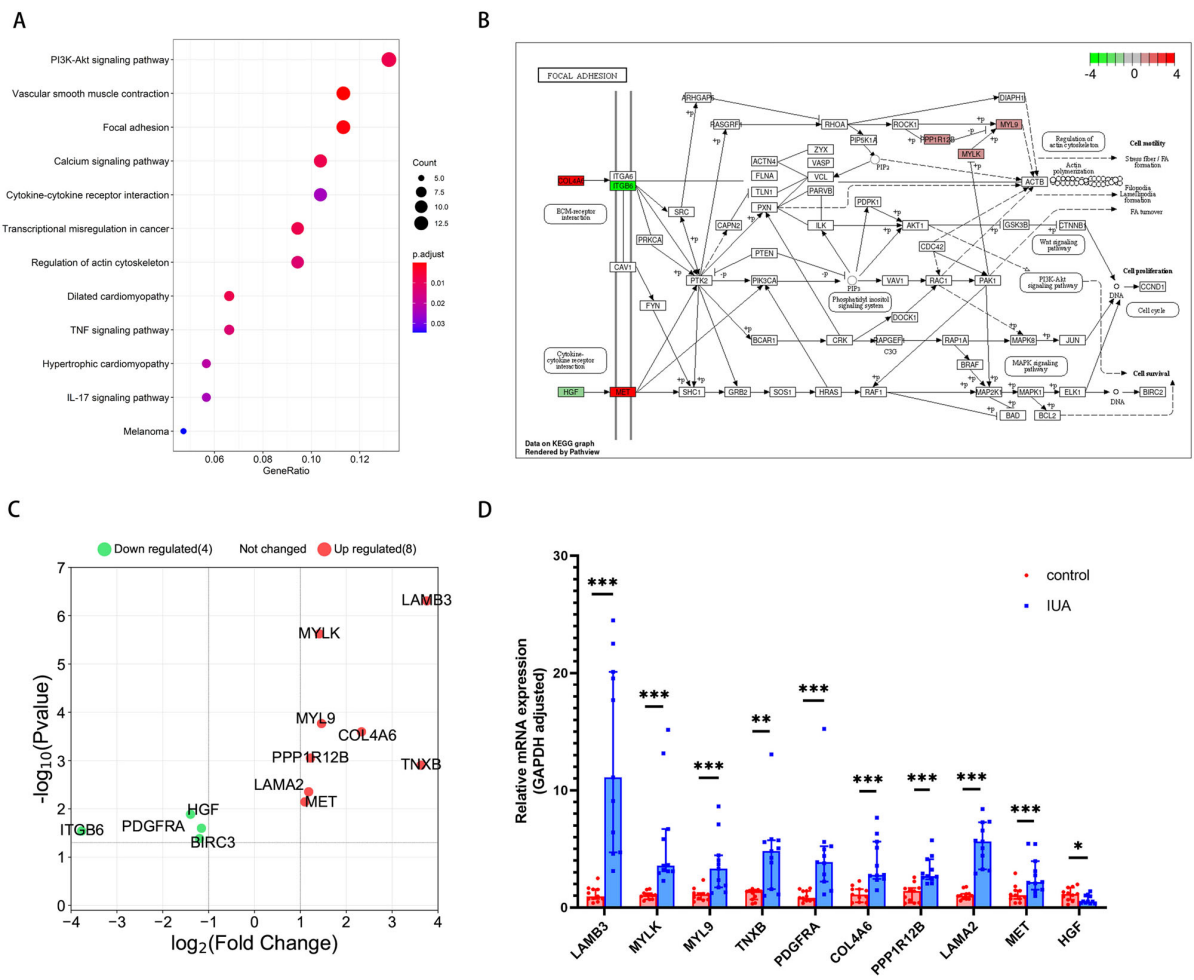
KEGG analysis of the differentially expressed genes (DEGs). **A** The top 12 regulated signaling pathways obtained from KEGG enrichment analysis in the IUA group; **B** KEGG analysis of the DEGs associated with the focal adhesion signaling pathway. The red and green rectangles represent the upregulated and downregulated genes, respectively; **C** The DEGs enriched in focal adhesion pathway were screened based on *p* value ≤ 0.05 and | Fold change | ≥ 2 at the same time; **D** Determinations of the mRNA expression levels of DEGs which enriching in focal adhesion pathway of the Control and IUA groups by qRT-PCR. *p value < 0.05. **p value < 0.01. ***p value < 0.001. ns: not significant

We used the David online database (https://david.ncifcrf.gov/summary.jsp) to conduct KEGG enrichment analysis for the 399 DEGs. The most significantly enriched pathways included the PI3K/AKT signaling pathway and those associated with vascular smooth muscle contraction and focal adhesion (Fig. 2A). The differential genes were obtained by KEGG enrichment analysis (Supplementary Table 3).The KEGG pathway map (Fig. 2B) also exhibited some cell effects such as stress fibers, which conforms to our hypothesis that these are involved with IUA. We then validated the reliability of the DEGs enriched in the focal adhesion pathway, as a basis for our subsequent studies. The RNA-seq results showed that the mRNA expression levels of LAMB3 was 9.07 times higher in the IUA group when compared to normal endometrial tissues (Fig. 2C). The sequencing results of genes enriched in the focal adhesion pathway are given in Supplementary Table 4. Determinations of the mRNA expression levels of DEGs which were enriched in the focal adhesion pathway of the Control and IUA groups by qRT-PCR are shown Fig. 2D.

### QT-PCR and IHC of LAMB3 Expression in Endometrial Tissues

**Fig. 3:**
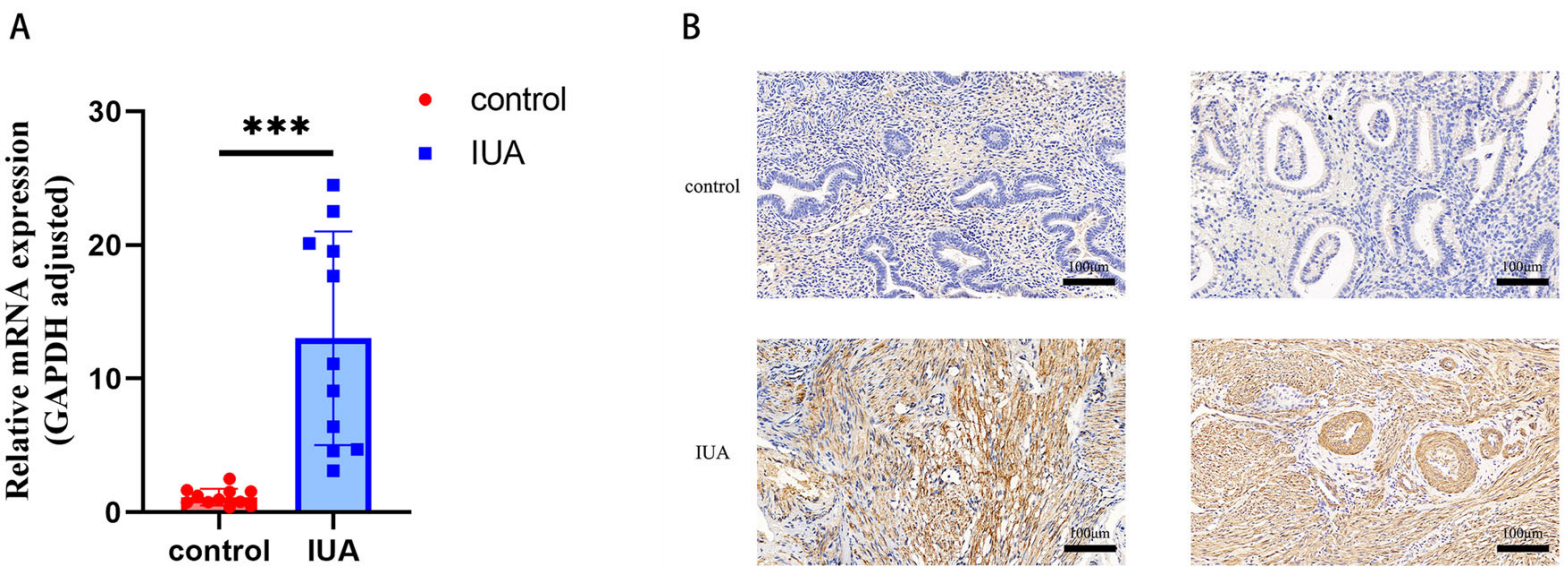
Expression of LAMB3 in endometrial tissue samples of patients with IUA. **A** The gene expression levels of LAMB3 in the endometrium of the Control and IUA groups as measured using RT-qPCR; **B** Representative IHC images of LAMB3 immuno-reactivity in IUA compared to normal endometrium tissues. GAPDH served as a loading control. *p value < 0.05. **p value < 0.01. ***p value < 0.001. ns: not significant

The results of qRT-PCR showed that the expression of LAMB3 mRNA in the endometrium of the IUA group was 14.06 times higher than that of normal endometrium (Fig. 3A). We used immunohistochemical staining to analyze the expression of LAMB3 protein in the endometrial tissues of patients in the IUA group and it was found to be higher than that of control group (Fig. 3B).

### LAMB3 Promoted Phenotypic Transformation of Myofibroblasts in ESCs

**Fig. 4:**
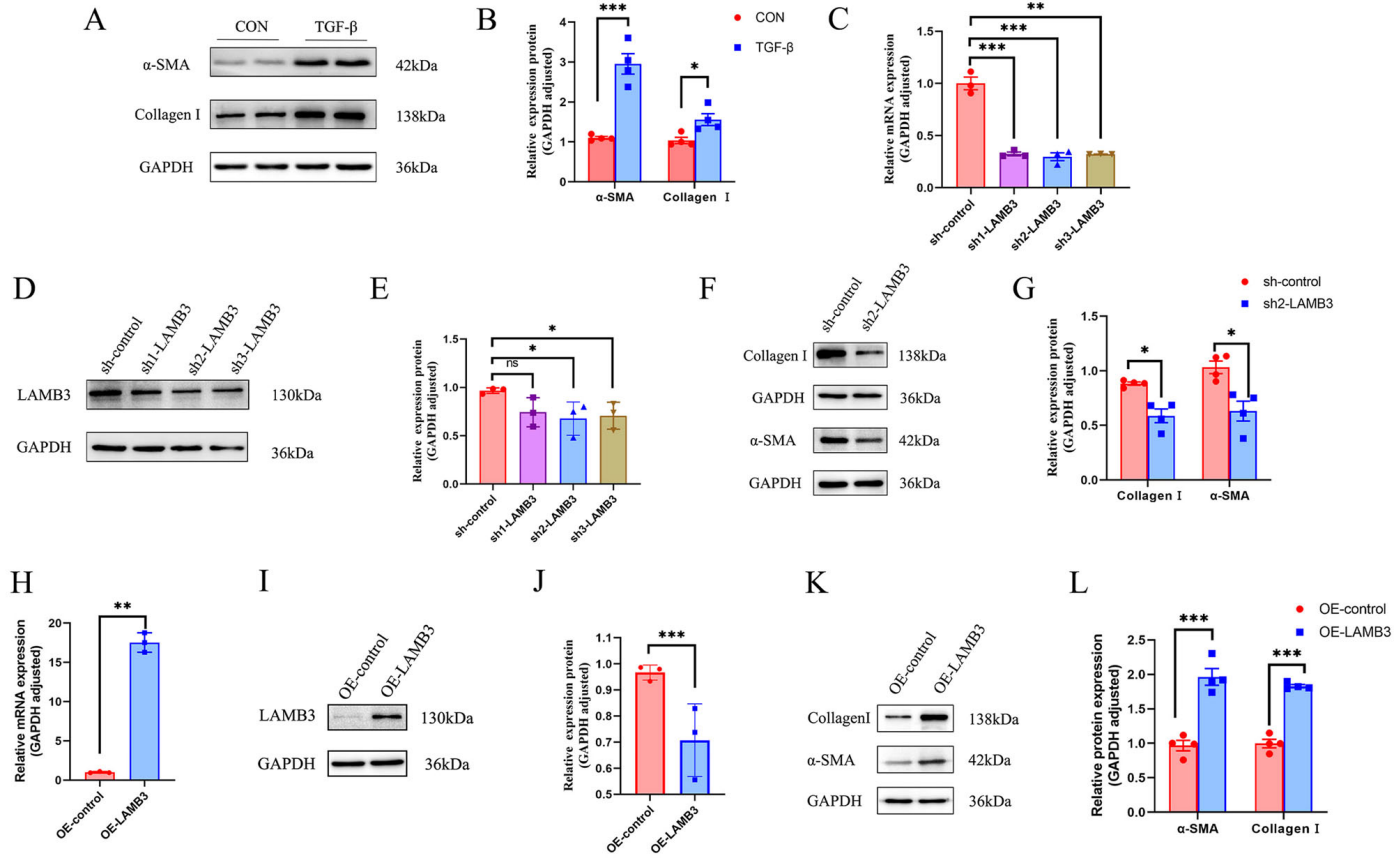
LAMB3 promoted the phenotypic transformation of myofibroblasts from endometrial stromal cells. **A**, **B** Gene expression levels of LAMB3 in CON and TGF-β in the endometrium measured using RT-qPCR. **C** LAMB3 mRNA expression levels were measured by qRT-PCR in sh-control and LAMB3-silenced (sh1-LAMB3, sh2-LAMB3) cells. **D**, **E** Western blotting analysis of LAMB3 protein expression levels in sh-control and LAMB3-silenced cells. **F**, **G** Western blotting analysis of collagen type I and α-SMA expression levels in sh-control and LAMB3-silenced cells. **H** LAMB3 mRNA expression was measured by qRT-PCR in OE-control and LAMB3-overexpressed (OE-LAMB3) cells. **I**, **J** Western blotting analysis LAMB3 protein expression levels in OE-control and LAMB3-overexpressed cells. **K**, **L** Western blotting analysis of collagen type I and α-SMA expression in OE-control and LAMB3-overexpressed cells. *p value < 0.05. **p value < 0.01. ***p value < 0.001. ns: not significant. Representative blots are shown in each case

Incubation of ESCs with 10 nM TGF-β for 48 h was used to construct an ESC myofibroblast model [27, 28]. Protein analysis verified that the protein expression levels of the myofibroblast markers, collagen type I and α-SMA, were both elevated after TGF-β treatment (Fig. 4A, B). In order to understand the role of LAMB3 in endometrial fibrosis, we silenced LAMB3 in primary cultures of human ESCs by using three shRNA constructs (sh1-LAMB3, sh2-LAMB3 and sh3-LAMB3) and confirmed that the gene was silenced through mRNA (Fig. 4C) and protein analysis (Fig. 4D, E). We then selected the sh2-LAMB3 cell model which produced the best silencing effect for further studies. Silencing of LAMB3 decreased the protein expression levels of collagen I and α-SMA in the ESC myofibroblast model (Fig. 4F, G). In addition, silencing of LAMB3 reversed the TGF-β-induced myofibroblast phenotype transformation of ESCs. We also overexpressed LAMB3 in ESCs (OE-LAMB3) (Fig. 4H–J), and confirmed that overexpression of this gene promoted myofibroblast phenotype transformation (Fig. 4K, L).

### LAMB3 Affects ESC Cytoskeleton Remodeling

**Fig. 5:**
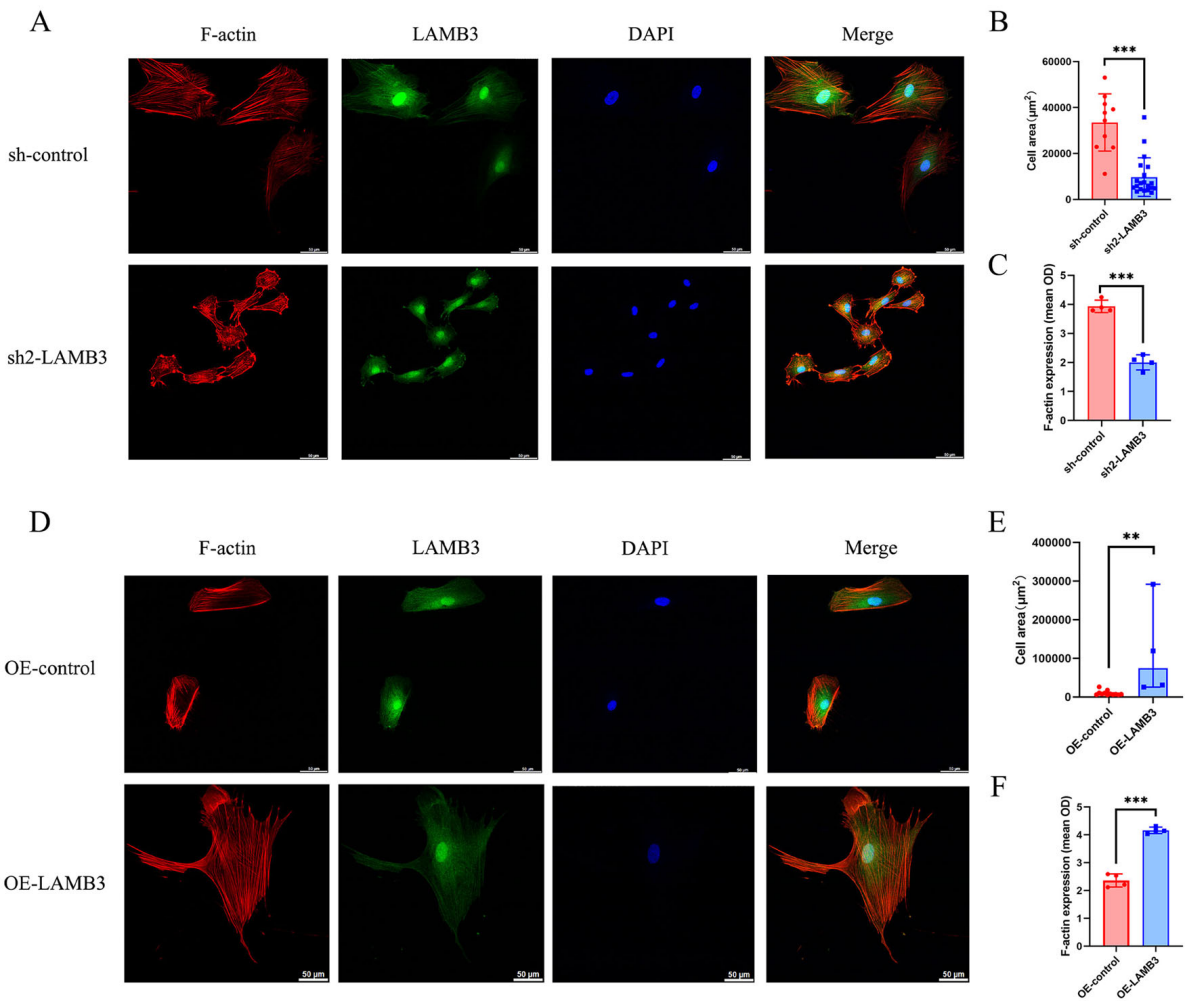
LAMB3 affected ESC cytoskeleton remodeling. **A** Representative confocal microscopy images of sh-control, and LAMB3-silenced (sh2-LAMB3) cells, stained with LAMB3 (green), phalloidin (red) and DAPI nuclear stain (blue). Scale bars = 50 µm. **B** Comparison of the average cell areas of ESCs between sh2-LAMB3 and sh-control cells. **C** Comparison of the average optical densities of ESCs between sh2-LAMB3 and sh-control. **D** Representative confocal microscopy images of OE-control and LAMB3-overexpressed (OE-LAMB3) cells. **E** Comparison of the average cell areas of OE-LAMB3 and OE-control. **F** Comparison of the average optical densities of ESCs between OE-LAMB3 and OE-control

Alpha-SMA can act as a molecular motor for cells to produce mechanical changes, with F-actin, which is a major component of the cytoskeleton, forming the scaffold [29–31]. Therefore, we investigated the effect of LAMB3 on the cytoskeletal remodeling of ESCs using phalloidin which specifically binds to F-actin and staining the cells after incubation. After transfection of sh2-LAMB3 into ESCs, the cell morphology was diverse, and many of the cells appeared to be star-like and have hammerhead-like shapes. Reorganization of cytoplasmic myosin was observed, with the arrangement of stress fibers appearing to be disorganized in a non-polar manner and they were unevenly distributed. Some cells exhibited dense concentrations at the edges (Fig. 5A). A comparison between the sh2-LAMB3 and sh-control groups revealed a decrease in the cell volume, optical density and the number of actin filaments in the ESCs (Fig. 5B, C). After overexpression of LAMB3, the ESCs became polygonal and enlarged, with visible pseudopodia, actin reorganization as well as abundant and thick filaments that were evenly distributed in the cytoplasm, showing stress fiber polarity (Fig. 5D). A comparison between overexpressed and control cells revealed an increased cell volume, optical density as well as increased numbers of actin filaments in the ESCs (Fig. 5E, F).

### Overexpression and Silencing of LAMB3 Activated The RhoA/ROCK1/MYL9 Signaling Pathway

**Fig. 6:**
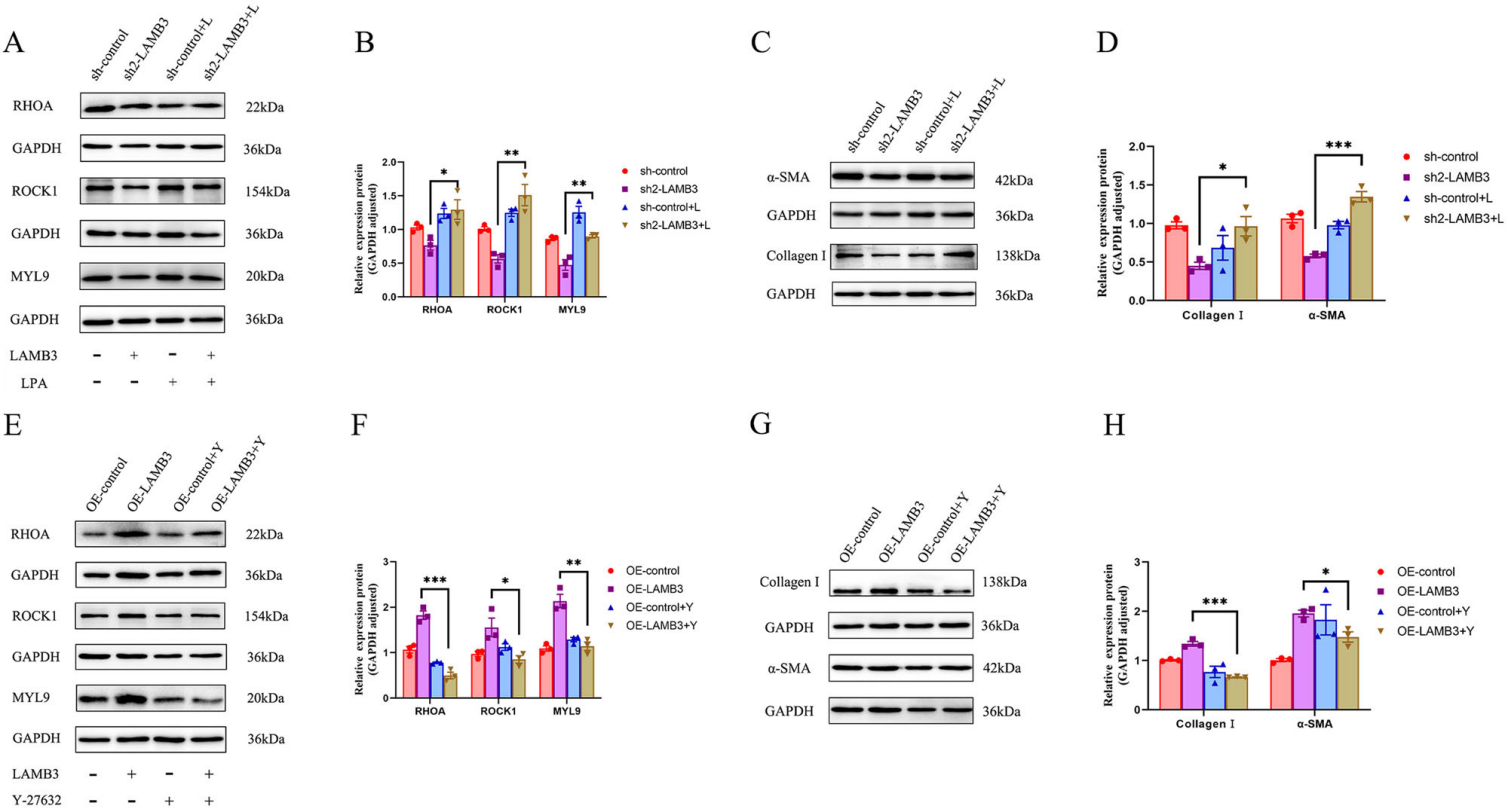
LAMB3 overexpression and silencing on the activation of the RhoA/ROCK1/MYL9 signaling pathway. **A**, **B** Western blotting analysis was used to measure the protein expression levels of Rho, ROCK1 and MYL9 in sh-control and LAMB3-silenced cells after stimulation with the activator, lysophosphatidic acid (LPA). **C**, **D** Western blotting analysis of collagen type I and α-SMA expression in sh-control and LAMB3-silenced cells stimulated by LPA. **E**, **F** Western blotting analysis was used to measure the protein expression levels of RhoA, ROCK1 and MYL9 in OE-control and LAMB3 overexpressed cells after incubation with the inhibitor, Y-27632. **G**, **H** Western blotting analysis of collagen type I and α-SMA expression in OE-control and LAMB3-overexpressed cells after addition of Y-27632. Representative blots are shown in each case

Network analysis identified the focal adhesion pathway as a basis for mechanistic investigations of IUA (Fig. 2A, C). RhoA, ROCK1 and MYL9 form a cascade of signal molecules in this pathway. Increasing evidence has suggested that RhoA and ROCK are involved with other signaling pathways to regulate mechanisms associated with fibrosis [32, 33]. We hypothesized that LAMB3 modulates the myofibroblastic phenotype transformation of ESCs through the RhoA/ROCK1/MYL9 pathway. We used the pathway activator, LPA, to restore inhibition of the RhoA/ROCK1/MYL9 signaling pathway and ESC myofibroblastic transformation by silencing LAMB3. The results showed that the protein expression levels of the signaling molecules were increased (Fig. 6A, B) and the myofibroblastic markers, collagen type I and α-SMA, were also upregulated (Fig. 6C, D). Using the pathway inhibitor, Y-27632, to restore the expression of LAMB3 had the opposite effects on the pathway and ESC myofibroblast differentiation (Fig. 6E–H).

## Discussion

AS is an acquired disorder characterized by the presence of intrauterine adhesions caused by injury of the basalis layer of the endometrium [34, 35]. This leads to a loss of the normal cyclic proliferation and secretions from the endometrium, and usually results in various adverse reproductive outcomes when the uterine cavity is blocked by scarred tissues [36, 37].

By using RNA-seq measurements, we found that the gene, LAMB3, was enriched in the focal adhesion pathway and its expression levels were significantly increased in patients with IUA when compared to those with normal endometrial tissue. LAMB3 is known to be involved in extracellular matrix deposition [35, 38] and it induces the overexpression of fibroblasts [39], making it a potential biomarker of invasion and metastasis. It is also a driving factor in wound healing and cancer invasion [40]. In terms of organ fibrosis, Lauritano et al. (2020) [39] demonstrated that LAMB3 was upregulated in immunosuppressant-treated myofibroblasts, where it promoted the accumulation of connective tissue. However, there have been no reports concerning the correlation between LAMB3 and endometrial fibrotic diseases.

We used in vitro cultures of ESCs to show that LAMB3 regulates their myofibroblastic transformation and cytoskeletal remodeling. However, we did not compare the phenotypes of cells derived from the uterus with those of cultured cells. We only compared their altered stimulatory phenotypes in the presence and absence of inducers. During tissue organ injury, myofibroblasts simultaneously secrete vast amounts of extracellular matrices and contract smooth muscle cells, thus repairing the defect and constricting the wound surface [41, 42]. An increase and/or a decrease in the extracellular matrix activates pro-fibrotic cytokines which disturbs the balance of degradation enzymes and this leads to excessive accumulation and deposition of interstitial collagen fibers. The fibers become cross-linked to form scarred tissue and eventually result in organ fibrosis changes [43, 44]. There is increasing evidence to suggest that RhoA/ROCK interact with other signaling pathways in order to regulate fibrosis [45]. RhoA belongs to the Rho small GTPase family which are known to regulate various mechanosensitive cellular functions, including cytoskeletal organization, cell polarity, proliferation and differentiation [46]. They can promote the formation and elongation of stress fibers and the contraction and directed adhesion of actin bundles [47].

This study verified the effects of silencing LAMB3 and overexpressing LAMB3 in ESCs through the RhoA/ROCK1/MYL9 pathway, as well as those of the activator, LPA, and the inhibitor, Y-27632. These experiments elucidated the role of LAMB3 in regulating ESC myofibroblastic transformation through the RhoA/ROCK1/MYL9 pathway. It also showed that Rho played an influential role in the epithelial-mesenchymal transition process [48, 49] and the production and transformation of α-SMA after induction by TGF-β [50], as well as the restructuring of the cellular skeleton [51]. This study has been able to relate the protein, LAMB3, and the RhoA/ROCK1/MYL9 pathway to endometrial fibrosis. These novel findings will help toward the potential screening of anti-endometrial fibrosis drugs and this could benefit patients with infertility in the future.

## Conclusions

**Fig. 7:**
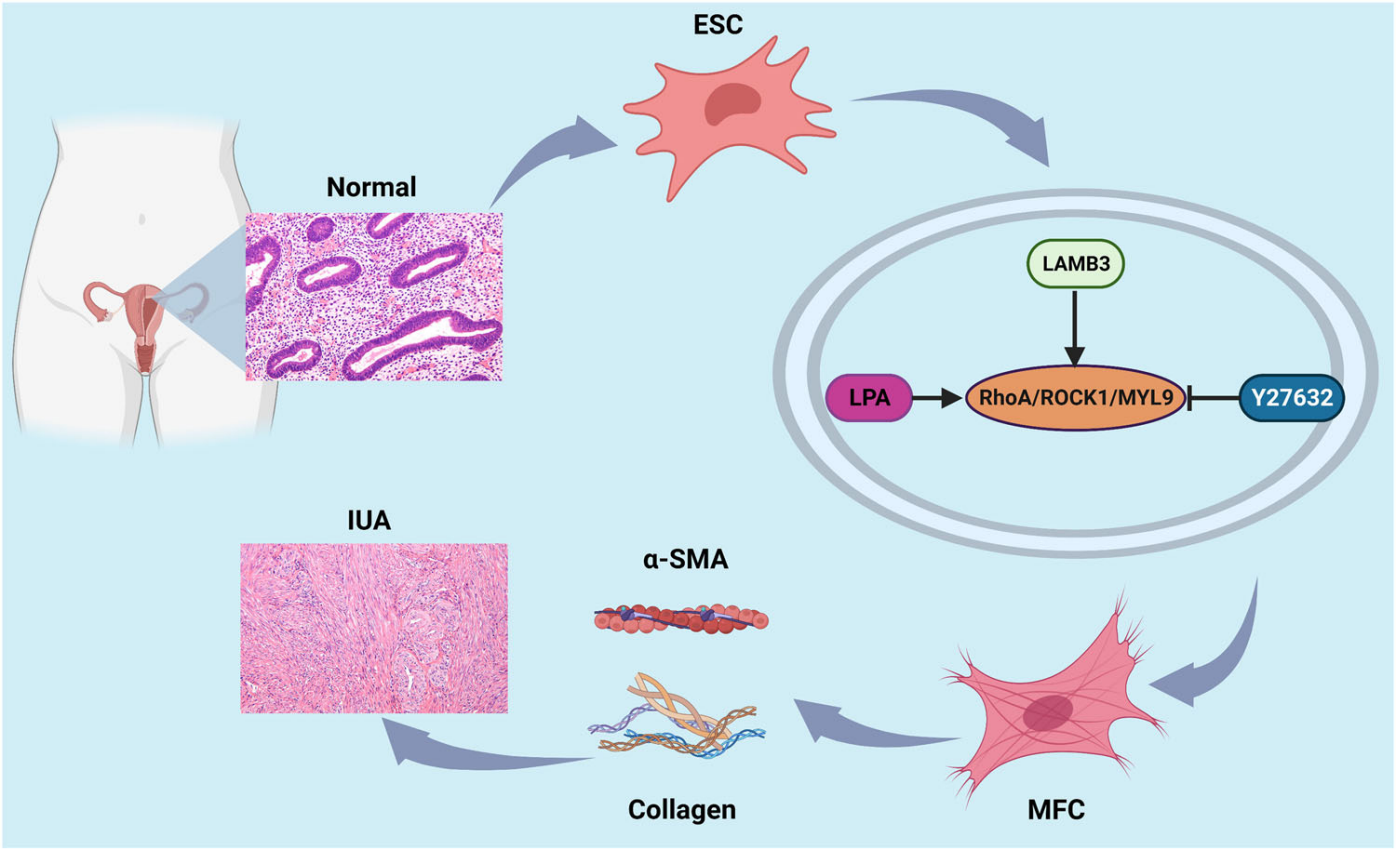
Summary of the mechanisms of the effect of LAMB3 on endometrial myofibrosis using an online drawing tool and element source: https://biorender.com/

This study demonstrated that LAMB3 can regulate the myofibroblastic transformation of ESCs through the RhoA/ROCK1/MYL9 pathway. We suggest that during myometrial fibrosis, LAMB3 via the RhoA/ROCK1/MYL9 pathway, modulates the cells to undergo fibrotic transformation with the participation of SMA. This leads to an increase in collagen fiber deposition and smooth muscle actin secretion, resulting in fibrous adhesion hyperplasia as well as contraction of the surrounding tissues (Fig. 7). However, the pathogenesis of adhesion in the cavity is complex, and there is currently no effective anti-fibrotic drug treatment. Therefore, this study provides a potential new target for therapeutic intervention which may help patients with AS.

### Supplementary information

Supplementary Figure 1

Supplementary Figure 2

Supplementary Figure 3

Supplementary Table 1

Supplementary Table 2

Supplementary Table 3

Supplementary Table 4

## Abbreviations

LAMB3: The β subunit of laminin-binding protein
RhoA: Ras homolog gene family, member A
ROCK1: Rho associated kinase
MYL9: Myosin light chain 9
ESC: Endometrial stromal cell
PCR: Polymerase chain reaction
LPA: Lysophosphatidic acid
TGF-β: Transforming growth factor beta
AS: Asherman’s syndrome
α-SMA: Alpha-smooth muscle actin
IUA: Intrauterine adhesion
RT-qPCR: Real-time fluorescent quantitative PCR
IHC: Immunohistochemical
KEGG: Kyoto Encyclopedia of Genes and Genomes
DEGs: Differentially expressed genes
RNA-seq: Ribonucleic Acid sequencing
