# Supplementary material for: LAMB3 Promotes Myofibrogenesis and Cytoskeletal Reorganization in Endometrial Stromal Cells via the RhoA/ROCK1/MYL9 Pathway
Source: Cell Biochem Biophys. 2023 Oct 6;82(1):127–37. doi: 10.1007/s12013-023-01186-5 (PMC10867058; doi:10.1007/s12013-023-01186-5)
Supplement: Supplementary file 1 — Supplementary Figure 1 [file 12013_2023_1186_MOESM1_ESM.pdf]

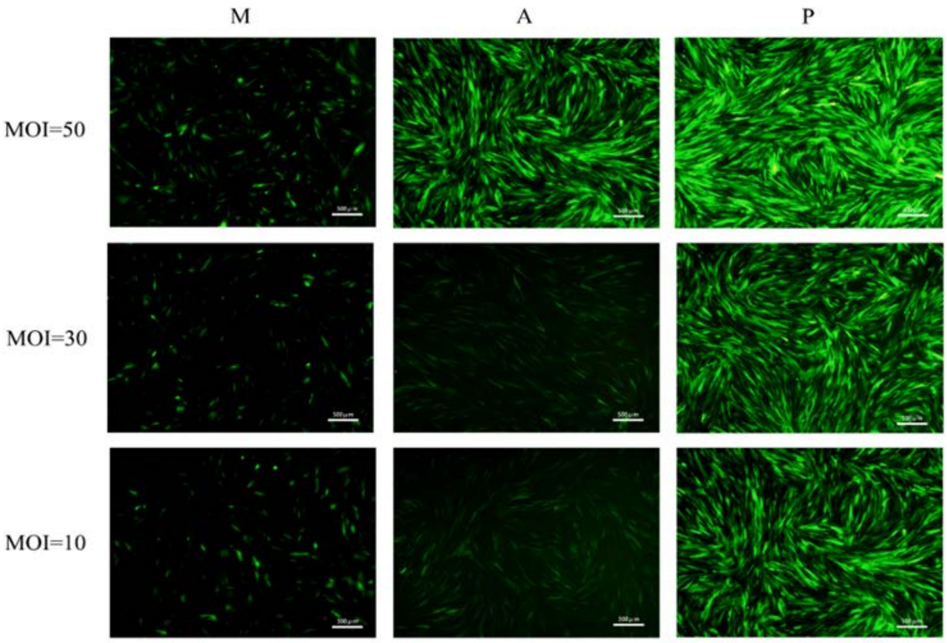

Supplementary Figure 1. Pre-transfection of ESCs.M = virus without the transfection enhancer, A = transfection enhancer + viruses, P = transfection enhancer + virus, and MOI stands for the infectivity of virus to cells. (Scale bars =50 µm; X100 mag).
