# Supplementary figures and images for: LAMB3 Promotes Myofibrogenesis and Cytoskeletal Reorganization in Endometrial Stromal Cells via the RhoA/ROCK1/MYL9 Pathway
Source: Cell Biochem Biophys. 2023 Oct 6;82(1):127–37. doi: 10.1007/s12013-023-01186-5 (PMC10867058; doi:10.1007/s12013-023-01186-5)

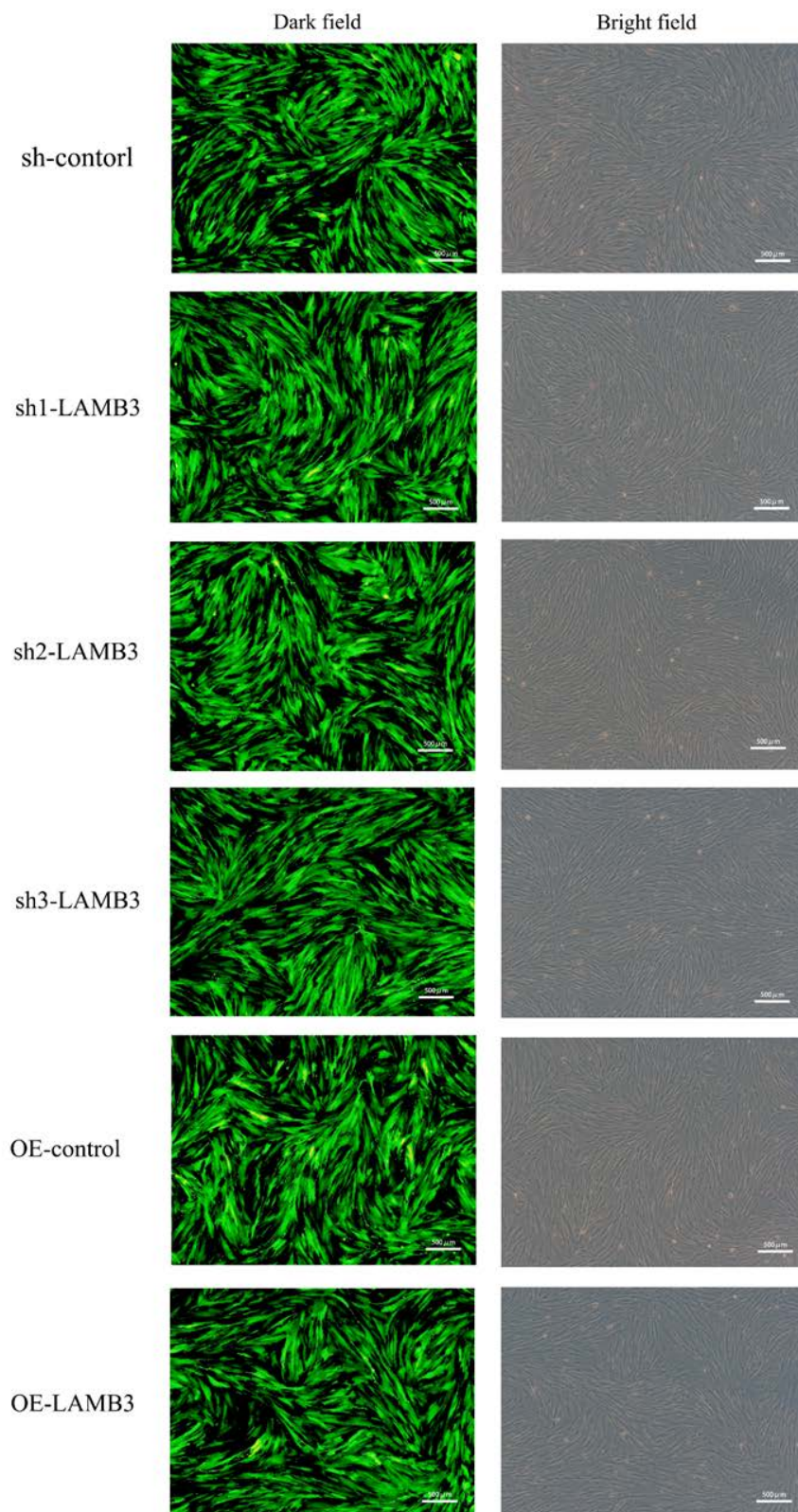

**Supplementary Figure 2.** Post-transfection of ESCs.(Scale bars =50  $\mu$ m; X100 mag).

Supplement: Supplementary file 2 — Supplementary Figure 2 [file 12013_2023_1186_MOESM2_ESM.pdf]
