# Supplementary material for: LAMB3 Promotes Myofibrogenesis and Cytoskeletal Reorganization in Endometrial Stromal Cells via the RhoA/ROCK1/MYL9 Pathway
Source: Cell Biochem Biophys. 2023 Oct 6;82(1):127–37. doi: 10.1007/s12013-023-01186-5 (PMC10867058; doi:10.1007/s12013-023-01186-5)
Supplement: Supplementary file 3 — Supplementary Figure 3 [file 12013_2023_1186_MOESM3_ESM.pdf]

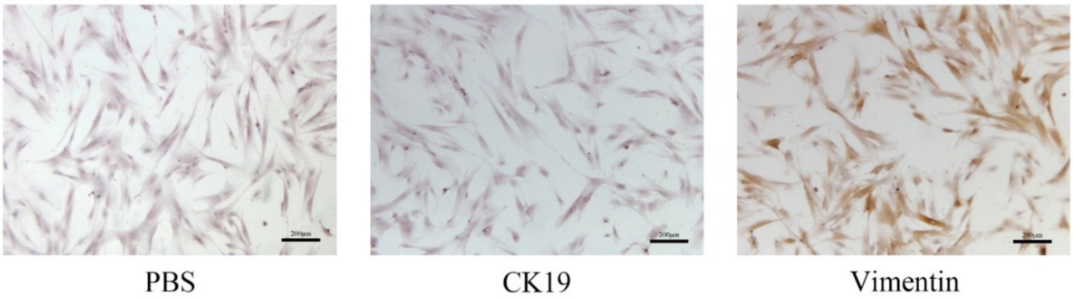

Supplementary Figure 3. Identification of ESCs using immunocytochemistry.

PBS represent refers to the negative control group, CK19 represents cytokeratin 19 staining to identify glandular epithelial cells, and vimentin represents vimentin staining to identify interstitial cells. (Scale bars =50 μm; X100 mag).
