# Supplementary material for: LAMB3 Promotes Myofibrogenesis and Cytoskeletal Reorganization in Endometrial Stromal Cells via the RhoA/ROCK1/MYL9 Pathway
Source: Cell Biochem Biophys. 2023 Oct 6;82(1):127–37. doi: 10.1007/s12013-023-01186-5 (PMC10867058; doi:10.1007/s12013-023-01186-5)
Supplement: Supplementary file 4 — Supplementary Table 1 [file 12013_2023_1186_MOESM4_ESM.pdf]

**Supplementary Table 1** Sequence information with respect to the lentiviruses used in this study.

| Sequence | Sequence Information |
|---|---|
| RNAi-LAMB3-1 | ccggccAAGAGGGATTTGAGAGAATctcgagATTCTCTCAAATCCCTCTTggtttttg |
| RNAi-LAMB3-2 | ccgggaAGCTTCAATGGTCTCCTTActcgagTAAGGAGACCATTGAAGCTtcttttg |
| RNAi-LAMB3-3 | ccgggaTCACAAACTTGAGAGTCAActcgagTTGACTCTCAAGTTTGTGAtcttttg |
| OV-LAMB3 | ATGAGACCATTCTTCCTCTTGTGTTTTGCCCTGCCTGGCCTCCTG<br>CATGCCCAACAAGCCTGCTCCCGTGGGGCCTGCTATCCACCTGT<br>TGGGGACCTGCTTGTTGGGAGGACCCGGTTTCTCCGAGCTTCAT<br>CTACTTGTGGACTGACCAAGCCTGAGACCTACTGCACCCAGTA<br>TGGCGAGTGGCAGATGAAATGCTGCAAGTGTGACTCCAGGCAG<br>CCTCACAACTACTACAGTCACCGAGTAGAGAATGTGGCTTCAT<br>CCTCCGGCCCCATGCGCTGGTGGCAGTCACAGAATGATGTGAA<br>CCCTGTCTCTCTGCAGCTGGACCTGGACAGGAGATTCCAGCTTC<br>AAGAAGTCATGATGGAGTTCCAGGGGCCCATGCCCGCCGGCAT<br>GCTGATTGAGCGCTCCTCAGACTTCGGTAAGACCTGGCGAGTG<br>TACCAGTACCTGGCTGCCGACTGCACCTCCACCTTCCCTCGGGT<br>CCGCCAGGGTCGGCCTCAGAGCTGGCAGGATGTTCGGTGCCAG<br>TCCCTGCCTCAGAGGCCTAATGCACGCCTAAATGGGGGGAAGG<br>TCCAACTTAACCTTATGGATTTAGTGTCTGGGATTCCAGCAACT<br>CAAAGTCAAAAAATTCAAGAGGTGGGGGAGATCACAAACTTG<br>AGAGTCAATTTCACCAGGCTGGCCcCTGTGCCCCAAAGGGGCT<br>ACCACCCTCCCAGCGCCTACTATGCTGTGTCCCAGCTCCGTCTG<br>CAGGGGAGCTGCTTCTGTCACGGCCATGCTGATCGCTGCGCAC<br>CCAAGCCTGGGGCCTCTGCAGGCCCCTCCACCGCTGTGCAGGT<br>CCACGATGTCTGTGTCTGCCAGCACAACACTGCCGGCCCAAAT<br>TGTGAGCGCTGTGCACCCTTCTACAACAACCGGCCCTGGAGAC<br>CGGCGGAGGGCCAGGACGCCCATGAATGCCAAAGGTGCGACT<br>GCAATGGGCACTCAGAGACATGTCACTTTGACCCCGCTGTGTTT<br>GCCGCCAGCCAGGGGGCATATGGAGGTGTGTGTGACAATTGCC<br>GGGACCACACCGAAGGCAAGAACTGTGAGCGGTGTCAGCTGC<br>ACTATTTCCGGAACCGGCGCCCGGGAGCTTCCATTCAGGAGAC<br>CTGCATCTCCTGCGAGTGTGATCCGGATGGGGCAGTGCCAGGG<br>GCTCCCTGTGACCCAGTGACCGGGCAGTGTGTGTGCAAGGAGC |

ATGTGCAGGGAGAGCGCTGTGACCTATGCAAGCCGGGCTTCAC
TGGACTCACCTACGCCAACCCGCAGGGCTGCCACCGCTGTGAC
TGCAACATCCTGGGGTCCCGGAGGGACATGCCGTGTGACGAGG
AGAGTGGGCGCTGCCTTTGTCTGCCCAACGTGGTGGGTCCCAA
ATGTGACCAGTGTGCTCCCTACCACTGGAAGCTGGCCAGTGGC
CAGGGCTGTGAACCGTGTGCCTGCGACCCGCACAACTCCCTCA
GCCCACAGTGCAACCAGTTCACAGGGCAGTGCCCCTGTCGGGA
AGGCTTTGGTGGCCTGATGTGCAGCGCTGCAGCCATCCGCCAG
TGTCCAGACCGGACCTATGGAGAC
GTGGCCACAGGATGCCGAGCCTGTGACTGTGATTTCCGGGGAA
CAGAGGGCCCGGGCTGCGACAAGGCATCAGGCCGCTGCCTCTG
CCGCCCTGGCTTGACCGGGCCCCGCTGTGACCAGTGCCAGCGA
GGCTACTGCAATCGCTACCCGGTGTGCGTGGCCTGCCACCCTTG
CTTCCAGACCTATGATGCGGACCTCCGGGAGCAGGCCCTGCGC
TTTGGTAGACTCCGCAATGCCACCGCCAGCCTGTGGTCAGGGC
CTGGGCTGGAGGACCGTGGCCTGGCCTCCCGGATCCTAGATGC
AAAGAGTAAGATTGAGCAGATCCGAGCAGTTCTCAGCAGCCCC
GCAGTCACAGAGCAGGAGGTGGCTCAGGTGGCCAGTGCCATCC
TCTCCCTCAGGCGAACTCTCCAGGGCCTGCAGCTGGATCTGCCC
CTGGAGGAGGAGACGTTGTCCCTTCCGAGAGACCTGGAGAGTC
TTGACAGAAGCTTCAATGGTCTCCTTACTATGTATCAGAGGAA
GAGGGAGCAGTTTGAAAAAATAAGCAGTGCTGATCCTTCAGGA
GCCTTCCGGATGCTGAGCACAGCCTACGAGCAGTCAGCCCAGG
CTGCTCAGCAGGTCTCCGACAGCTCGCGCCTTTTGGACCAGCTC
AGGGACAGCCGGAGAGAGGCAGAGAGGCTGGTGCGGCAGGCG
GGAGGAGGAGGAGGCACCGGCAGCCCCAAGCTTGTGGCCCTG
AGGCTGGAGATGTCTTCGTTGCCTGACCTGACACCCACCTTCAA
CAAGCTCTGTGGCAACTCCAGGCAGATGGCTTGCACCCCAATA
TCATGCCCTGGTGAGCTATGTCCCCAAGACAATGGCACAGCCT
GTGGCTCCCGCTGCAGGGGTGTCCTTCCCAGGGCCGGTGGGGC
CTTCTTGATGGCGGGGCAGGTGGCTGAGCAGCTGCGGGGCTTC
AATGCCCAGCTCCAGCGGACCAGGCAGATGATTAGGGCAGCCG
AGGAATCTGCCTCACAGATTCAATCCAGTGCCCAGCGCTTGGA
GACCCAGGTGAGCGCCAGCCGCTCCCAGATGGAGGAAGATGTC
AGACGCACACGGCTCCTGATCCAGCAGGTCCGGGACTTCCTAA
CAGACCCCGACACTGATGCAGCCACTATCCAGGAGGTCAGCGA
GGCCGTGCTGGCCCTGTGGCTGCCCACAGACTCAGCTACTGTTC
TGCAGAAGATGAATGAGATCCAGGCCATTGCAGCCAGGCTCCC

CAACGTGGACTTGGTGCTGTCCCAGACCAAGCAGGACATTGCG
CGTGCCCGCCGGTTGCAGGCTGAGGCTGAGGAAGCCAGGAGCC
GAGCCCATGCAGTGGAGGGCCAGGTGGAAGATGTGGTTGGGA
ACCTGCGGCAGGGGACAGTGGCACTGCAGGAAGCTCAGGACA
CCATGCAAGGCACCAGCCGCTCCCTTCGGCTTATCCAGGACAG
GGTTGCTGAGGTTCAGCAGGTACTGCGGCCAGCAGAAAAGCTG
GTGACAAGCATGACCAAGCAGCTGGGTGACTTCTGGACACGGA
TGGAGGAGCTCCGCCACCAAGCCCGGCAGCAGGGGGCAGAGG
CAGTCCAGGCCCAGCAGCTTGCGGAAGGTGCCAGCGAGCAGG
CATTGAGTGCCCAAGAGGGATTTGAGAGAATAAAACAAAAGT
ATGCTGAGTTGAAGGACCGGTTGGGTCAGAGTTCCATGCTGGG
TGAGCAGGGTGCCCGGATCCAGAGTGTGAAGACAGAGGCAGA
GGAGCTGTTTGGGGAGACCATGGAGATGATGGACAGGATGAA
AGACATGGAGTTGGAGCTGCTGCGGGGCAGCCAGGCCATCATG
CTGCGCTCGGCGGACCTGACAGGACTGGAGAAGCGTGTGGAGC
AGATCCGTGACCACATCAATGGGCGCGTGCTCTACTATGCCAC
CTGCAAG
