# Supplementary material for: LAMB3 Promotes Myofibrogenesis and Cytoskeletal Reorganization in Endometrial Stromal Cells via the RhoA/ROCK1/MYL9 Pathway
Source: Cell Biochem Biophys. 2023 Oct 6;82(1):127–37. doi: 10.1007/s12013-023-01186-5 (PMC10867058; doi:10.1007/s12013-023-01186-5)
Supplement: Supplementary file 5 — Supplementary Table 2 [file 12013_2023_1186_MOESM5_ESM.pdf]

Supplementary Table 2 The primer sequences used in this study.

Primer name	Primer sequence (5'-3')
LAMB3-F	CTTATGGATTTAGTGTCTGGG
LAMB3-R	CTGGTGAAATTGACTCTCAAG
MYLK-F	CTGCTGCCTGACCACGAATA
MYLK-R	GGTAATCAACCTCGGGCTCC
MYL9-F	CTTCACAGATGAGGAAGTGGAC
MYL9-R	TGAACTCCACGTAGTTGAAGTT
COL4A6-F	CCATCCTCGAATCAAGGCGA
COL4A6-R	GGCTCACCTCTCATGCCTTT
PPP1R12B-F	TCACGGAGCCAGTGTAGGTA
PPP1R12B-R	TTCCCACTGTTGAGCCACTG
TNXB-F	AACCCCGTATGAGCTGTCAC
TNXB-R	GAGGACTTCCCAGGCTTCTC
LAMA2-F	AATAAATCTCGCTGTGAGTG
LAMA2-R	GTTAGAAAAGTTCCAGCTCTC
MET-F	ATCAGGAGGTGTTTGGAAAGAT
MET-R	CGACTGTATGTCAGCAGTATGA
HGF-F	AATCCACTCATTCCTTGGGATT
HGF-R	TCCCATTTACAACTCGCAATTG
PDGFRA-F	GAAAATGAAAAGGTTGTGCAGC
PDGFRA-R	CTCTTCTTCAGACATGGGGTAC
COL1A1-F	TTTCTGCCCGTTGGGCTTAT
COL1A1-R	ATTGCCTTTGATTGCTGGGC
α-SMA-F	GGCTCTGGGCTCTGTAAGG
α-SMA-R	CTCTTGCTCTGGGCTTCATC
LAMB3-F	CTTATGGATTTAGTGTCTGGG
LAMB3-R	CTGGTGAAATTGACTCTCAAG
GAPDH-F	CAGGAGGCATTGCTGATGAT
GAPDH-R	GAAGGCTGGGGCTCATTT
