# Supplementary material for: LAMB3 Promotes Myofibrogenesis and Cytoskeletal Reorganization in Endometrial Stromal Cells via the RhoA/ROCK1/MYL9 Pathway
Source: Cell Biochem Biophys. 2023 Oct 6;82(1):127–37. doi: 10.1007/s12013-023-01186-5 (PMC10867058; doi:10.1007/s12013-023-01186-5)
Supplement: Supplementary file 6 — Supplementary Table 3 [file 12013_2023_1186_MOESM6_ESM.pdf]

Supplementary Table 3 The differential genes obtained by KEGG enrichment analysis.

Gene Name	Count	Fold-change	*p*-value	FDR
Vascular smooth muscle contraction	13	3.3	6.40E-05	1.50E-02
AGE-RAGE signaling pathway in diabetic complications	10	2.5	5.10E-04	5.60E-02
Focal adhesion	14	3.5	8.10E-04	5.60E-02
Hypertrophic cardiomyopathy	9	2.3	1.10E-03	5.60E-02
TNF signaling pathway	10	2.5	1.20E-03	5.60E-02
IL-17 signaling pathway	9	2.3	1.50E-03	5.90E-02
Dilated cardiomyopathy	9	2.3	1.70E-03	5.90E-02
Calcium signaling pathway	14	3.5	3.90E-03	1.10E-01
Transcriptional misregulation in cancer	12	3	5.20E-03	1.10E-01
cAMP signaling pathway	13	3.3	5.40E-03	1.10E-01
Rheumatoid arthritis	8	2	5.80E-03	1.10E-01
Pathways in cancer	23	5.8	5.90E-03	1.10E-01
