# Supplementary material for: LAMB3 Promotes Myofibrogenesis and Cytoskeletal Reorganization in Endometrial Stromal Cells via the RhoA/ROCK1/MYL9 Pathway
Source: Cell Biochem Biophys. 2023 Oct 6;82(1):127–37. doi: 10.1007/s12013-023-01186-5 (PMC10867058; doi:10.1007/s12013-023-01186-5)
Supplement: Supplementary file 7 — Supplementary Table 4 [file 12013_2023_1186_MOESM7_ESM.pdf]

Supplementary Table 4 The sequencing results of genes enriched in the focal adhesion pathway.

Gene Name	log_2FC	*p-value*
LAMB3	3.753701818	0.000000493
MYLK	1.411318943	0.00000238
MYL9	1.459961592	0.000171088
COL4A6	2.331421725	0.00025411
PPP1R12B	1.218375515	0.000887069
TNXB	3.629513714	0.001248304
LAMA2	1.179929019	0.004431394
MET	1.097109359	0.00717125
HGF	-1.395215589	0.012949226
PDGFRA	-1.157035995	0.025229454
ITGB6	-3.769200269	0.028776975
BIRC3	-1.200069167	0.041263724
